# Preparation and Microcosmic Structural Analysis of Recording Coating on Inkjet Printing Media

**DOI:** 10.3390/ijms12085422

**Published:** 2011-08-23

**Authors:** Bo Jiang, Weiyan Liu, Yongping Bai, Yudong Huang, Li Liu, Jianping Han

**Affiliations:** Polymer Materials and Engineering Department, School of Chemical Engineering and Technology, Harbin Institute of Technology, P.O. Box 410, Harbin 150001, China; E-Mails: jiangbo_5981@yahoo.com (B.J.); wy_liu@sohu.com (W.L.); bai_yp@yahoo.com.cn (Y.B.); liu_li70@yahoo.com.cn (L.L.); han_j_p@yahoo.com.cn (J.H.)

**Keywords:** recording coating, inkjet printing, analysis

## Abstract

Preparation of recording coating on inkjet printing (RC-IJP) media was proposed. The microstructure and roughness of RC-IJP was analyzed by scanning electron microscopy (SEM) and atomic force microscope (AFM). The surface infiltration process of RC-IJP was studied by a liquid infiltration instrument. The distribution of C, O and Si composites on recording coating surface is analyzed by energy dispersive spectrum (EDS). The transmission electron microscopy (TEM) analysis showed that the nanoscale silica could be dissolved uniformly in water. Finally, the print color is shown clearly by the preparative recording coating.

## 1. Introduction

With the growth of the inkjet market, consumer interest in inkjet printing material, being its main consumable product, has increased. Inkjet printing has been reviewed in organic [1,2], papermaking [3,4] and nanotechnology [5] industries, *etc*. P. Calvert [6] has summarized current inkjet technology and survey applications in materials of organic transistors, light emitting diodes, ceramics, and biopolymer arrays. B. Derby [7] has reviewed the current state of understanding of the mechanisms of drop formation and defines the fluid properties.

The recording coating on inkjet printing (RC-IJP) has a distinct effect on the printing quality. The distribution of the particles in the recording coating determines the infiltration of ink. The dispersibility of the recording coating is more uniform in terms of good ink infiltration and is poorer in terms of long infiltration time; this result induces the ink diffusion on the recording coating. In order to enhance the printing quality, the preparation of RC-IJP is very important.

According to our knowledge from the literature, focus has mainly been on inkjet printing technology, ink drop formation and applications of the materials. However, preparation and microcosmic structural analysis of the recording coating on the ink jet printing (RC-IJP) has been less studied less until now. The aim of this study is therefore the preparation and microcosmic structural analysis of RC-IJP. In order to enhance the printing quality, the dispersibility of the nanoscale silica in the recording coating is studied.

## 2. Results and Discussion

### 2.1. The Microcosmic Structure of RC-IJP

**Figure 1.**
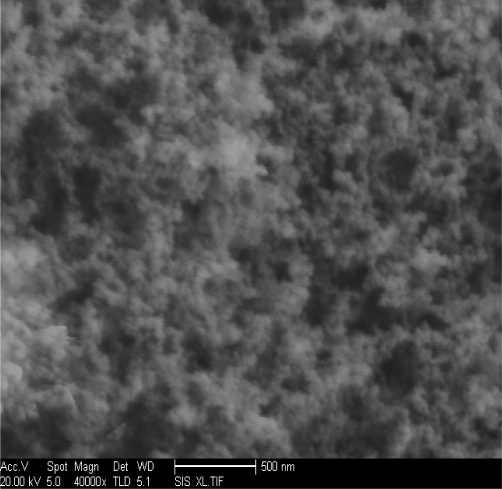
Scanning electron microscopy (SEM) of the nanoscale silica.

**Figure 2.**
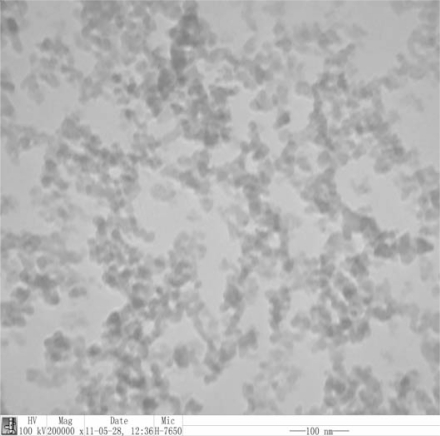
Transmission electron microscopy (TEM) of the nanoscale silica in the solution.

Scanning electron microscopy (SEM) of the nanoscale silica is shown in Figure 1. Transmission electron microscopy (TEM) of the dispersibility of the nanoscale silica in the solution is shown in Figure 2. The average particle size of the nanoscale silica is 12 nm in the experiment, the particle size of the nanoscale silica after disjection did not obviously change. The dispersibility of the nanoscale silica in the solution is uniform as shown in Figure 2.

**Figure 3.**
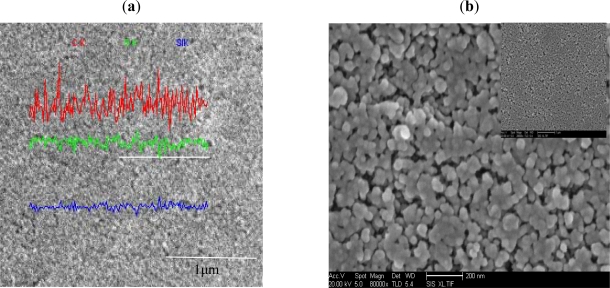
Surface of recording coating on inkjet printing (RC-IJP): (**a**) Morphologies of SEM; and (**b**) Distribution of C, O and Si composites by EDS.

SEM of RC-IJP is shown in Figure 3(a), the surface RC-IJP is smooth and the dispersibility of the nanoscale silica in RC-IJP is uniform. This result is advantageous for ink infiltration. The distribution of the main element composition (C, O and Si) on recording coating surface is shown in Figure 3(b) by energy dispersive spectrum (EDS). The analytical result implies that dispersibility of each composition is uniform in the recording coating.

**Figure 4.**
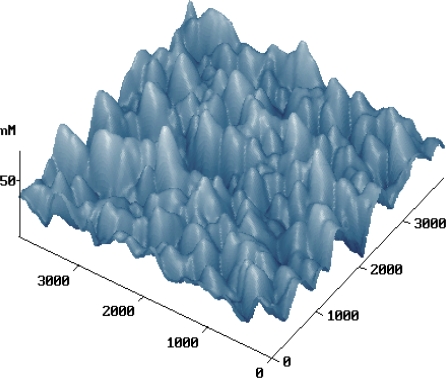
Atomic force microscope (AFM) image of RC-IJP.

Microcosmic features of the RC-IJP surface was analyzed by atomic force microscope and is shown in Figure 4. The surface of RC-IJP has a number of heaves, which benefit ink adsorption. On the other side, the dispersibility of the roughness is uniform as seen from Figure 4, preventing the ink from diffusing.

### 2.2. The Surface Infiltration of RC-IJP

In order to evaluate RC-IJP, the surface infiltration time and the color of the printing image are the most important indexes. If the weight of per unit area recording material is invariant, the dispersibility of the recording coating is more uniform in terms of the short surface infiltration time. The color of the printing image is clearer by preparative RC-IJP.

**Figure 5.**
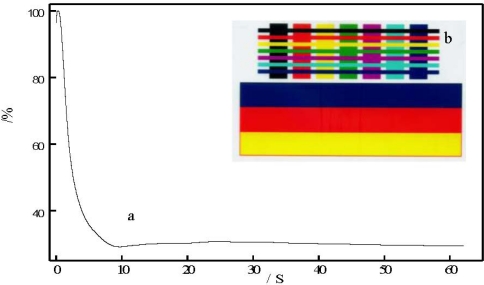
RC-IJP: (**a**) surface infiltration time; and (**b**) the color of the printing image.

Figure 5 shows the infiltration time of recording coating surface. In this Figure 5(a), x and y axes show the infiltration time of the sample and the energy of ultrasound, respectively. Figure 5(a) shows the rate at which water infiltrates on recording coating surface, being 0.20 s, while simultaneously RC-IJP receives full energy (100%) under ultrasound. In Figure 5(b), the color of the printing image is clear.

## 3. Experimental Section

### 3.1. Materials

**Figure 6.**
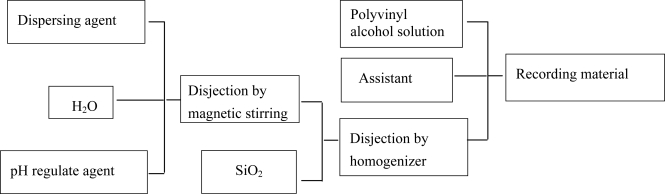
Preparation process of the recording coating.

The inorganic pigments-nanoscale silica (the average particle size is 12 nm, specific surface area is 170–230 m^2^/g) obtained from Degussa A200, Germany. The binder-polyvinyl alcohol (PVA-1788), the substrate-polyethylene terephthalate (PET) and accessory ingredient were provided by Shanghai Chemical Co., Ltd, China. The recording coating prepared is shown in Figure 6.

Silicane dispersing agent (γ-aminopropyltriethoxysilane) is added into distilled water under magnetic stirring for 10 min, nitric acid is subsequently added until solution pH = 4 by ultrasound 10 min. The nanoscale silica is added in the above solution under homogenizer (Fluko Equipment Shanghai Co., Ltd.) for 30 min. The weight scale of the silicane dispersing agent, nanoscale silica and distilled water is 0.02:1:5, respectively. The dispersive nanoscale silica solution is added in 8% PVA solution (distilled water as solvent) by homogenizer for 10 min, a little ethanol (as control assistant of solution viscosity) is added in mixed solution. Finally, the mixed homogeneous solution is added and placed in 40 °C oven for 120 min.

### 3.2. Characterization

The dispersibility of the nanoscale silica in the solution is analyzed by transmission electron microscopy (TEM, FEI, Quanta 200F, made in the USA). The surface morphologies of RC-IJP are observed using a high-resolution scanning electron microscopy (SEM, Quanta 200 F, made in the USA). The surface roughness of RC-IJP is examined by atomic force microscope (AFM, Solver-P47H, NT-MDT, Russia). The surface infiltration time which is analyzed by liquid infiltration instrument (Emtec EST12 Co., Ltd., Germany) is the time that water infiltrates on the recording coating surface under ultrasound.

## 4. Conclusions

The above results showed that the distribution of the particle size is uniform in RC-IJP and the color of the printing image is clear. The preparation of RC-IJP was achieved using a green, facile, low-cost method which could easily be applied for industrial manufacture.
